# Measuring State and Trait Anxiety: An Application of Multidimensional Item Response Theory

**DOI:** 10.3390/bs13080628

**Published:** 2023-07-28

**Authors:** Leonardo Carlucci, Marco Innamorati, Melissa Ree, Giorgia D’Ignazio, Michela Balsamo

**Affiliations:** 1 Learning Science Hub, University of Foggia, 71121 Foggia, Italy; 2 Department of Human Sciences, Università Europea di Roma, 00163 Rome, Italy; 3 School of Psychological Science, University of Western Australia, Perth 6009, Australia; 4 Department of Psychological, Health and Territorial Sciences, University of “G. d’Annunzio” Chieti-Pescara, 66100 Chieti, Italy

**Keywords:** anxiety, psychometrics, somatic, cognitive, assessment, bifactor

## Abstract

The State-Trait Inventory for Cognitive and Somatic Anxiety (STICSA) is a widely used measure of state and trait anxiety. Within the Classical Testing Theory model, consistent findings provide support for its multidimensional factor structure, discriminant, convergent, and nomological validity, as well as age and gender invariance, across healthy and clinical samples. Nevertheless, some issues regarding STICSA dimensionality and item-scale composition remain unresolved (e.g., both bifactor and two-factor models were found to fit data equally well). The goal of this study was to investigate the STICSA’s dimensionality within the Item Response Theory, and to assess the tenability of the bifactor model as a plausible model over the multidimensional model. The sample consisted of 3338 Italian participants (58.21% females; 41.79% males) with an average age of 35.65 years (range: 18–99; SD = 20.25). Both bifactor and two-correlated dimensions of the STICSA scales were confirmed to fit data by applying the multidimensional Item Response Theory (mIRT). While the bifactor model showed better fit indices, the multidimensional model was more accurate and precise (0.86–0.88) in estimating state and trait latent anxiety. A further comparison between multidimensional item parameters revealed that the multidimensional and bifactor models were equivalent. Findings showed that the STICSA is an accurate and precise instrument for measuring somatic and cognitive symptomatology dimensions within state and trait anxiety. The use of the state/trait total score requires special attention from the clinicians and researchers to avoid bias in the psychodiagnostic assessment.

## 1. Introduction

Anxiety has a hypothetical multidimensional nature and multiple symptomatic manifestations [1,2]. Anxiety is multidimensional as it can be divided into different categories, including trait and state anxiety and both cognitive and somatic components (e.g., Martens, Vealey [3] proposed the multidimensional anxiety theory). This is a challenge to researchers and clinicians interested in the assessment of this complex phenomenon [4]. The main concern is the dimensionality of anxiety [1,2]. Similar to other psychological states, anxiety is considered to be composed of state and trait dimensions. It is possible to differentiate between transient emotion that varies in duration and is characterized by observable symptoms, like worry, tension, nervousness, and arousal of the autonomic nervous system (i.e., state anxiety), as well as a more enduring unobservable tendency to typically respond with high levels of anxious apprehension to perceived threats (i.e., trait anxiety) [5,6]. 

State anxiety has been conceptualized as an emotional response experienced in a limited period, depending on the anticipation of a (real or perceived) threatening stimulus, and is variable in duration [7]. On the other hand, trait anxiety has been viewed as an individual difference regarding an emotional response [8]. It is relatively stable in time and corresponds to neuroticism [9] and negative or low emotionality [10], is a risk factor for the development of anxiety disorder [11], and is comparable to anxiety sensitivity [12]. Individuals with high state anxiety may experience increased levels of apprehension, fear, and physiological arousal [13]. Conversely, individuals high in trait anxiety become more state anxious (in terms of duration, frequency, and intensity of the episodes of state anxiety) in critical contexts than those low in trait anxiety [7]. 

Within the assessment of self-reported anxiety, the state–trait distinction has been captured by distinct instructions for the state and trait scale (e.g., “generally” vs “right now”) by many authors [14,15]. Both state and trait anxiety have somatic and cognitive elements, creating four distinct components of anxiety: somatic state anxiety, somatic trait anxiety, cognitive state anxiety, and cognitive trait anxiety [13]. Clinicians and researchers have devoted much attention to the differentiation between somatic and cognitive anxiety [16]. From the clinical point of view, the symptoms of anxiety involve a broad range of cognitive, physical, and emotional aspects (e.g., “numbness,” “unsteadiness,” and “feeling hot”) that may include worry, intrusive thoughts, and lack of concentration [17]. This somatic/cognitive distinction might allow for a more fine-grained assessment of anxiety and facilitating clinical treatments to be tailored to the specific and predominant manifestation of anxiety symptomatology (e.g., physiologically oriented relaxation vs cognitively oriented) [16].

A controversial issue in assessing anxiety concerns its overlap with depression [18,19,20]. In line with the tripartite model of anxiety and depression [21], a negative effect (e.g., fear, anger, guilt) is associated with both anxiety and depression; the lack of positive effect (e.g., feeling tired) is related to depression, whereas physiological hyperarousal (e.g., trembling, dizziness, shaking) is associated with anxiety [22]. Thus, the overlap between anxiety and depression may lead to misdiagnosis, a fairly frequent problem in clinical settings [23]. To this end, the State–Trait Inventory for Cognitive and Somatic Anxiety (STICSA) [17] represents a multidimensional measure of state/trait and somatic/cognitive anxiety, which holds strong discriminant power with depression [24,25,26]. 

Previous research supported its multidimensional factor structure (of both state and trait STICSA scales, with each including somatic and cognitive dimensions) [27]. Additionally, it has displayed gender and age invariance, as well as good convergent validity with concurrent anxiety measures and sound internal discriminant validity as determined in large non-clinical samples [27,28,29,30,31,32]. Whilst the STICSA state and trait scales have been recently reaffirmed as independent measures each composed of somatic and cognitive dimensions [27], the STICSA’s dimensionality is still an open issue [33,34]. In detail, fitting both confirmatory (CFA) and exploratory structural equation models (ESEM) to STICSA scores, Styck and colleagues [33] showed that the oblique two-factor and bifactor models fit their data equally well for the separate state and trait forms of the STICSA. They also concluded that cognitive and somatic factors were not equally robust and that STICSA items appear to measure a mixture of both latent somatic and cognitive anxiety. Styck, Rodriguez [33] highlighted that “at least three items appear to meaningfully load onto both somatic-cognitive factors”, which complicates STICSA score interpretation (p. 21). The items “feel agonized over problems” (#3), “trouble remembering things” (#11), and “feel trembly and shaky” (#8) were found to cross-load onto both somatic and cognitive dimensions. However, this datum is trivial since the magnitude of these cross-loadings was below the recommended level of = 0.30 [35] on the non-target dimension [33]. In addition, the differences among the primary and alternative factor loadings were found to be above the recommended cut-off of 0.20 [36], except for item #11.

Taken together, these results were not surprising, since under certain statistical conditions, the correlated factors and bifactor models were covariance matrix-nested [37], and resulted in being statistically indistinguishable [38]. In this case, the implied covariance matrix of the STICSA-State/Trait cognitive-somatic correlated factors model can be perfectly reproduced by the bifactor model. In addition, it is well established that the bifactor model has a high fitting propensity (the “probifactor bias” [39]). Equally, cross-loadings might arise because of the similar content of indicators when measuring closely related but not identical latent variables [40]. Furthermore, cross-loadings’ magnitude is strictly related to the sample size and the type of the rotation applied in factor analysis [41]. 

Additionally, Styck, Rodriguez and Yi [33] affirmed that “STICSA state-trait cognitive and somatic anxiety composite scores do not purely measure latent somatic and cognitive anxiety” both in past and recent studies [17,27,34,42], and “that researchers must make a choice between competing alternatives that is appropriate for their particular study aims” (p.22). As a result, the authors did not provide clear guidance about the dimensionality of the STICSA, suggesting to directly model data complexity through a more flexible and exploratory approach, such as the ESEM. Indeed, the main core of their study shifted from comparing the competing models (bifactorial vs two-factor model) to the impossibility of distinguishing somatic from cognitive items domain in each state and trait scale. Despite the complexity of the data and the use of multiple techniques that fall within the classical testing theory (CTT) framework (i.e., the use in tandem of CFA and ESEM [33]), some issues regarding STICSA dimensionality and item-scale composition remain unresolved. Firstly, if STICSA items measure a nonnegligible mixture of both latent somatic and cognitive anxiety, each state and trait scale should be best represented by a single latent dimension or by the global dimension that arises from the bifactor model. Secondly, the computation of a total state/trait scale score would result in an accurate and precise estimation of anxiety. In addition, if fit indices cannot be used to select championed competing models [33], how can we assess the tenability of a somatic and cognitive specific dimension and its items in a specific model compared to others? A different psychometric approach to the STICSA dimensionality assessment would lead to interesting results. 

Hence, a step forward in the analysis of the STICSA state and trait scale could be represented by the application of modern psychometrics approaches, i.e., the Item Response Theory (IRT). One of the most important differences between CTT and IRT is that in CTT, one uses a common estimate of the measurement precision that is assumed to be equal for all individuals. Differently, in IRT, the measurement precision depends on the latent-attribute value (θ) [43]. The IRT was also found to be superior to CTT in individual change detection (especially when tests included at least 20 items) [43]. Next, assessing dimensionality, addressing items’ local independency issue, and estimating item parameters and information (e.g., difficulty and discrimination) under IRT assumptions could lead to important benefits in health/clinical assessments [44,45]. The IRT approach is particularly useful for the development and refinement of clinical measures since it allows the researcher to place the abilities of both respondents and items along a single continuum and measure them with common metrics. For example, IRT can capture meaningful nuances in clinical test accuracy since clinical measures are not equally reliable for all intervals of latent-attribute distributions. Again, IRT could allow clinical psychologists to replace total scores with more accurate IRT-based latent-attribute estimates (test scoring) [45]. 

However, no research has been conducted to assess STICSA dimensionality and properties using IRT models. The IRT models have always been conceived and estimated as unidimensional (or one-dimensional) models (e.g., the Rasch model) due to their attractiveness and measurement properties. However, unidimensional models are often simple and fail to fit the data when more items measure multiple latent traits simultaneously [46]. In addition, clinical instruments were found to provide unusual results about item parameters in clinical applications of IRT models, resulting in violations of the unidimensional and invariance IRT assumptions (for analogy, see the debate in intelligence using Jensen’s method; i.e., [47]). In the latter case, the estimation of a multidimensional IRT (mIRT) model is more appropriate [48]. Far from unidimensional IRT models, the estimation of multidimensional IRT models (e.g., bifactor mIRT; two- or three- dimensional mIRT models) may help to address the complex items issue, which involve multiple latent traits [48]. 

For example, estimating the STICSA bifactor mIRT model could allow for the measurement of a global anxiety dimension (technically labeled as latent-trait or latent-attribute [49]), along with the specific dimensions (e.g., somatic and cognitive), each representing distinct underlying concepts. Compared to the bifactor model estimated within a CTT framework, the bifactor mIRT model could provide full-information item analysis estimating item parameters. Thus, the mIRT models may represent a feasible methodological approach that allows to evaluate complex psychological constructs that might be understood as a combination of nested sub-scale components (compensatory factors) or a more general construct (non-compensatory factors) [48]. On the other hand, using the general dimension slope parameter to interpret the relationship between the item and its dimension may be confusing and misleading, since the probability of response in one dimension is conditioned by the other dimensions [50].

Based on the above, our main goal was to assess the tenability of the bifactor model as a plausible model that may explain each STICSA state/trait scale over the two-factor model [33]. Next, a comparison at the item parameter level was made by estimating the discrimination and difficulty indices. We examined the dimensionality of the STICSA via IRT since recent advancements, such as the multidimensional IRT, may aid researchers in disentangling the debate on its structure and items functioning, as well as to provide suggestions for improving the psychometric qualities of this measure. 

## 2. Materials and Method

### 2.1. Participants

The sample was recruited through advertisements in many Italian cities; the majority of the sample was taken from a published database [27] to which new datasets from unpublished studies have been added (N = 256). The STICSA was administered into the Italian language by licensed psychologists. Socio-demographic variables (such as age, gender, and education) were also collected. Inclusion criteria were ages from 18 to 99 years and the ability to complete self-administered questionnaires. Signed informed consent was obtained before the administration. The ethics committee of the Department of Psychological Sciences, Health and Territory, University of Chieti, Italy, approved the study.

### 2.2. Measures

The State–Trait Inventory for Cognitive and Somatic Anxiety (STICSA; [17]) (for the Italian version, see [27,31,32]) is a 21-item measure designed to assess cognitive and somatic symptoms, both on state and trait. In the state anxiety scale, they rate how they feel at the moment of assessment (from 1- “not at all” to 4- “very much”), whereas in the trait anxiety scale, participants rate how often a statement is true in general (from 1- “almost never at all” to 4- “almost always”).

### 2.3. Statistical Analysis

Preliminarily, IRT assumptions of uni-dimensionality of the STICSA state and trait were assessed using non-parametric IRT Mokken analysis [51,52]. All analyses were conducted using the mIRT package in R [53]. The Automated Item Selection Procedure (AISP) algorithm implemented in the Mokken package of R, using the recommended range of values of c = 0.3–0.5, has been used to partition a set of items (or a set of unscalable items) into Mokken scales [54,55]. The Mokken scale is defined by a set of dichotomously (e.g., yes/no) or polytomous (e.g., 1–4 Likert type response scale) scored items for which all inter-item covariances are positive and scalability coefficient (Hi for a single item/H for the total scale) values < 0.4 identify weak scalability; values between 0.4 and 0.5 are evaluated as moderate, and values > 0.5 as strong scalability [54]. Next, local independence to test the assumption of residual relationship amount item responses, that are not accounted for by the unidimensional model, was assessed by the standardized LD-χ2 statistic [56]. Large values of standardized LD residuals (equal to |10| or greater) reflected LI issues. LD-χ2 statistics were calculated using the residuals function in the mIRT package of R. A violation of both uni-dimensionality and local independence assumptions determined the non-appropriateness of a single factor model and the need for a hierarchical model (and thus a multidimensional approach).

According to the STICSA polytomous response format, the graded response model (GRM) and its multidimensional logistic model (mGRM) [48,57] was applied to evaluate the monotonicity of item response function (e.g., estimating difficulty and discrimination in the item parameters) on model(s) selected. In order to test the adequacy of the models, we computed the C2* fit statistic [58] available using the M2 function implemented in the mIRT package. The C2* is a limited-information goodness of fit test statistic for ordinal IRT models. Following the debate in literature on the STICSA, a series of competing models were also addressed (described below). The goodness of fit of the GRM models was also evaluated based on several fit statistics, such as the comparative fit index (CFI), the Tucker-Lewis index (TLI), the root mean square error of approximation (RMSEA), and the standardized root mean square residual (SRMR), that are strictly related to those in confirmatory factor analysis [59]. The −2*Likelihood (−2LL; which is distributed as Chi-square with degree of freedom) with the provided Aikake Information Criterion (AIC) [60] and Bayesian Information Criterion (BIC) [61] were used to select the most parsimonious model among the competing ones. The model with the lower −2LL and AIC/BIC score is expected to strike a superior balance between its ability to fit the data set and its ability to avoid over-fitting the data set. 

The item diagnostic was assessed using the signed χ2 test (S-χ2) [62]. A significance test (*p* < 0.05) was used under the null hypothesis to select misfitting items. Given that large samples and a large family of tests tend to yield significant χ2 values, the Benjamini and Hochberg [63] procedure was applied to adjust the obtained *p*-values for the number of tests in the family and to control for experimenter-wise error. 

For the unidimensional model, we estimated single slope (discrimination) parameters and three intercept (c) parameters for each 4-category STICSA item. Discrimination parameter values theoretically range from ±∞ (0.5 to 3.0 is a reasonable range). Intercept parameter (b) values ranging from −3 to + 3 are in the typical range [64]. For hierarchical and multidimensional IRT models, Multidimensional Item Discrimination (MDISC) and Multidimensional Difficulty Index (MDIFF) were also computed, transforming the estimated slope (item discrimination) and intercept parameters (category threshold). Values of MDISC >0.65 indicate discriminative items, and high positive values of MDIFF (>0.5) indicate difficult items [49,65]. Test information function (TIF) and related standard errors of measurement (SE) indicating the precision of the whole test were also estimated to determine at what level of the STICSA state and trait provide the most information. 

#### Models Tested

First, a unidimensional structure (Model 1-uni-GR) was examined with all 21 items defining one latent dimension for the separate trait and state forms of the STICSA. The single factor model was evaluated to assess if a unified concept underlying the data exists, as well as to evaluate the suitability of the multidimensional IRT assumption. 

Next, Model 2 (multi-GR) tested the original conceptualization of the STICSA (i.e., the oblique somatic-cognitive dimensions). This model is in line with recent studies suggesting that each STICSA form is well-represented by two correlated factors—cognitive and somatic anxiety [17,26,27,33,42,66]. 

Model 3 tested a bifactor mIRT model (bifac-GR) for the separate state and trait forms of the STICSA. In the bifactor model, each STICSA state/trait item was restricted to define both a global anxiety factor (i.e., global state anxiety; global trait anxiety) as well as a domain-specific factor (somatic or cognitive) [33,67]. In the bifactor mIRT model, somatic and cognitive domains for each state and trait scale were treated as orthogonal (uncorrelated), and item discrimination was estimated only in the dimension they belonged to, with the discrimination parameters linking items to the remaining dimensions being constrained to zero. 

Besides the above, a restricted bifactor IRT measurement model was modeled to address IRT assumption violations (i.e., local independency), which can turn up in a biased estimation of item parameters and/or an overestimation of test reliability [68]. A trace lines transformation (also named “marginal slopes”) [50,69] was applied to the bifactor mIRT model response function weighting by the normal distribution and integrating out the specific dimension and other general dimensions. 

Next, we applied a pairwise comparison strategy of item fit and parameter estimates and their total information/precision across (a) the uni-GR vs bifac-GR item; and (b) the multi-GR vs bifac-GR. The first comparison was done in order to assess the usefulness of a general bifactor model, as well as the presence of LD issues that reflected the presence of a multidimensional structure of the STICSA state/trait scales. 

Next, given the high degree of overlap among nested models, we compared multidimensional item parameters of multi-GR and bifac-GR models in order to assess the presence of meaningful differences among the models’ multidimensional parameters and the compensatory/non-compensatory nature of the STICSA state and trait scales. 

## 3. Results

### 3.1. Sample Characteristics

Recruited participants were 3338 Italian adults ranging from 18 to 99 years old, including 1943 females (58.21%) and 1395 males (41.79%), most of whom were undergraduate students. The mean age for the entire sample was 35.65 (SD = 20.25) years, compared to increasing Italian national average age (46 years) (ISTAT, 2021). The mean age for men was 37.78 (SD = 20.68) years, and 34.10 (SD = 19.79) years for females. Among these, 248 (7.4%) respondents held a primary school diploma, 461 (13.8%) a lower secondary school diploma, and 1966 (64.9%) a high school diploma, while 247 (10.4%) held a higher education (bachelor/master/doctorate) and the remaining (10.9%) declared no education or preferred not to answer. Concerning marital status, 2178 (65.2%) participants were unmarried/single, 801 (24%) were married, 108 (3.6%) were divorced/separated, 76 (2.3%) were cohabiting, 123 (3.7%) were widowed, and 52 (1.6%) did not answer.

### 3.2. Item Response Theory Assumptions

Preliminarily, we examined the frequency distribution of all item responses to identify items with high percentages at the extreme ends of the response scale. Such items could affect the subsequent stages of the analysis, especially the analyses conducted within the IRT framework [70]. The results showed that no items had a mean <0.5 and >3.5. From the total sample, we removed N = 13 cases with missing completely at random (MCAR) data on all the STICSA state and trait items. Cases with missing completely at random values were also retained in the IRT analyses. The final database was composed of 3,325 observations.

The STICSA state and trait scale items were submitted for Mokken analysis to test the uni-dimensionality assumption. The AISP revealed that all the STICSA state and trait items loaded on multiple latent dimensions, respectively. The inter-item covariances were found to be positive, and all the item scalability coefficients (Hi) ranged from 0.311 to 0.428 (weak) for the state, and from 0.295 to 0.404 (weak) for the trait scale; hence, the second criterion of the Mokken scale was not satisfied. The scalability coefficient for the entire state and trait scale, equal to 0.370 and 0.343, showed a weak scalability. As expected, the assumption of uni-dimensionality was not met for the 21 items of the state and trait scales.

Next, we assessed the tenability of local independence assumptions for each item pair of the state and trait scale, taking in to account the three models tested (see Appendix A). Large positive (+) and negative (−) standardized LD values for the models tested (uni-GR, multi-GR, and bifac-GR models) were summarized in Table 1. A careful examination of the LD values exhibited a pattern of 8/5 large positive LD among item pairs within the somatic area and 8/4 large positive LD among item pairs within the cognitive area regarding the state and trait scale, respectively. Far from the positive values, which can inflate the item slope parameter, the negative LD values can be ignored since they have a tendency to underestimate the slope parameter [71]. Negative LD values are usually detected for self-rating scales that do not measure best performance. The unmodeled covariation, particularly within the cognitive and somatic domains of state anxiety, suggested that a specific number of latent variables explaining the item response patterns or properly accounting for all covariations may not be outlined. This suggested that items for the state and trait scale may be better modelled by models defined as “multidimensional”, such as a two-correlated factors model or bifactor model. 

Therefore, a fit of the item response data with a multi-GR model and bifac-GR model was revealed. Again, by a detailed examination, unmodeled local dependency was found again in the LD values for the multidimensional model. However, it was isolated to three and four items in the somatic content area with five and two items in the cognitive content area for the state and trait scale, respectively. Fortunately, the adoption of a multi-GR model would appear to lead towards results that facilitate the local independence assumption. Whether or not the bifac-GR model was fitted to the item response data to better account for the unique variance attributed to each content area, the unmodeled LD was significantly reduced. The only LD values included items #1 and #16 for both the state and trait scale, with three other items (items #2, #12, and #10 for the state scale), and item #9 for the trait scale. The bifac-GR model resulted in the enhancement of the modelling of the item response covariations. This result leads to unique variability to be modelled for each content area after accounting for a general latent trait.

### 3.3. Models-Data Fit and Comparison

Table 1 summarizes the results of the model comparison and displays fit indexes for the several IRT models in the comparison. All indices for determining the best-fitting STICSA state-trait model seemed to agree with the selected model, which was the bifac-GR model. However, the goodness of fit for the multi-GR model indicated an acceptable-to-good fit for the sample, strictly close to the bifactor model (see CFI and TLI indices above the >0.95 threshold and a RMSEA ≤0.05 for excellent fit). Nevertheless, the deviance statistics (−2LL) and the absolute difference in AIC and BIC (>|9|) [72] provided support for the superiority of the bifactor solution over the multi-GR model. The goodness of fit of the uni-GR model confirmed a less good model fit for STICSA state-trait scales (see LD values).

### 3.4. Comparison of Item Fit and Parameters

The uni-GR model and bifac-GR model parameter estimates for the STICSA state and trait items, respectively, were summarized in Table 2. We examined fit at the item level for the uni- and bifac-GR models controlling for Type I error using a Benjamini-Hochberg adjustment. Concerning the uni-GR and bifac-GR models, none of the state scale items was identified as misfitting. On the contrary, item #14 (*p* = 0.004) for the trait scale was identified as misfitting in both models, using the same criteria.

Comparing the uni-GR with bifac-GR model solutions revealed a range from 0 to 0.99 with a mean absolute difference of 0.21 (SD = 0.29) for the trait scale, and a range from 0 to 3.04 of the absolute differences in the values of the item intercept parameters with a mean absolute difference of 0.39 (SD = 0.53) for the state scale, as Appendix B shows. In particular, the absolute value of the mean difference was larger for the third intercept at the extreme; that is, 0.36 (SD = 0.39) for the trait scale and 0.62 (SD = 0.70) for the state. The mean absolute difference was lower for the first intercepts, 0.14 (SD = 0.21) and 0.06 (SD = 0.05) for trait and state scales, respectively. On the other hand, the mean average absolute difference was elevated for the second intercept in the state scale [0.41(SD = 0.48)] compared to the trait scale [0.21(SD = 0.23)]. Indeed, by ignoring LD, the loss of precision created led to shrinkage in intercept values for extreme categories, as you can see in Appendix B. Inflating the conditional slopes of the general latent trait in the bifac-GR resulted in the conditional relationships among the specific and general latent traits [50]. Therefore, since a straight comparison among specific and general slopes is not recommended, a marginal slope [69] was computed by using Stucky and Edelen [50,69] equations and comparing slopes between the two model solutions (uni vs. bifac model).

Concerning the state scale, a comparison of the general state conditional slopes (see Table 2) from the bifac-GR model solution shows 10 of the 21 item conditional slopes were larger than uni-GR model slopes. The average absolute difference was 0.33 (Min = 0.09, Max = 1.67). For the general trait conditional slope, the bifac-GR model solution shows 11 of the 21 item conditional slopes were larger than uni-GR model slopes (see Table 2). The average absolute difference was 0.28 (Min = 0.06, Max = 0.96). An inspection of the marginal slopes in Table 2 shows that the four items which discriminated more across respondents with respect to the general state dimension, in rank order, were items 13 (a13*g = 3.932), 17 (a17*g = 2.909), 9 (a9*g= 2.882), 19 (a19*g = 2.597), and 3 (a3*g = 2.207). Two items discriminate more across respondents with respect to the general trait dimension: items #8 (a8*g = 2.349) and #14 (a14*g = 2.109). A comparison across marginal and conditional slopes of the bifac-GR models showed that there is a slight difference in the magnitude only for those items which have slopes close to 0 on the somatic dimension of the state scale, and negligible difference only for somatic items of the trait scale.

However, the marginal slopes for the general state and trait scales were much smaller in size for items which had a relevant conditional slope on the specific somatic/cognitive dimensions (i.e., state items #8 [a8_S1_ = 1.99], #14 [a14_S1_ = 1.92], and #18 [a18_S1_ = 1.77]; trait items #7 [a7_S1_ = 1.50] and #8 [a8_S1_ = 1.92]). 

### 3.5. Comparison of TIFs and Marginal Reliability 

**Figure 1:**
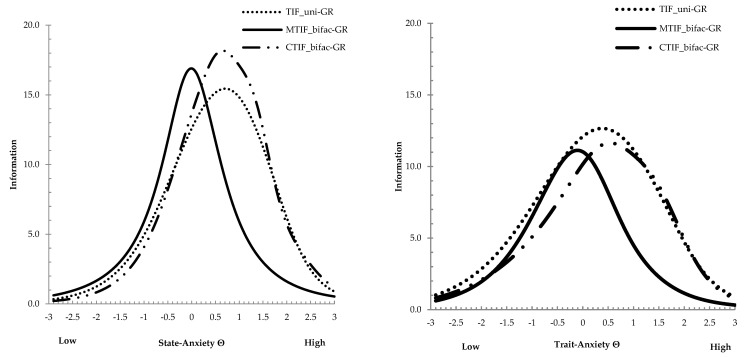
Total information function (TIF) for the state (**left**)/trait (**right**) STICSA. Note: TIF = unidimensional TIF; CTIF = conditional TIF fit by the bifactor GR model based on conditional slopes; MTIF = marginal TIF fit by the bifac−GR model based on marginal slopes. TIF with prior variance of 1 (posterior total [test] information).

**Figure 2:**
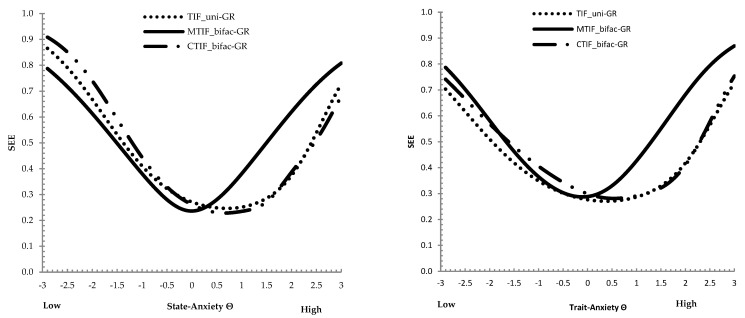
Standard Error of Estimate (SEE) for the state (**left**)/trait (**right**) STICSA. Note: TIF = unidimensional SEE; CTIF = conditional SEE fit by the bifactor GR model based on conditional slopes; MTIF = marginal SEE fit by the bifac−GR model based on marginal slopes.

Next, the total information functions were compared across the uni-GR and bifac-GR models for the STICSA state and trait scales. How well the construct is measured across any level of the trait continuum is the main information derived from using the TIFs. A straightforward comparison between the TIF from the unidimensional (uni-GR) model and the conditional bifac-GR (CTIF) model evidenced that the two functions seemed to overlap along all latent STICSA-state values (the conditional bifac-GR model was found to be more informative than uni-GR) (see Figure 1). Concerning the trait scale, the uni-GR was found to be more informative and included the conditional bifac-GR information that was slightly narrowed along the STICSA-trait values (see Figure 1). Based on the MTIF in Figure 1, the measurement precision is roughly constant for values on the state/trait continuum closest to 0, and between −0.5 and 0.5 with total information for the 21-item state/trait of about 15~14 (TIF with prior variance of 1—posterior total [test] information; 41~42 without prior variance of 1). The standard errors (SEEs; Figure 2) of bifac-GR state/trait scores for this interval were close at about 0.25~0.26 (1/√Information). This level of error around the scores converts to an IRT reliability value of about 1−SEE^2^, which was 0.93−0.94 in this interval, respectively, for the state and trait scales. 

Additionally, when the marginal reliability (also named empirically, since we used an EAP estimator) of the unidimensional model (0.89 for state, 0.90 for trait) and the bifactor model (0.87 for state, 0.86 for trait) was contrasted, there was an inflation in score precision when LD was overlooked (see Table 1). This inflated effect was more pronounced in the trait scale. On the other hand, the estimation of the conditional bifac-GR model tended to overestimate state/trait reliability/precision scores along the right segments of the continuum (high state/trait anxiety). Next, the marginal reliability for the two-correlated model (multi-GR, 0.86–0.88 for state and trait, for somatic and cognitive factors), respectively, was found to be higher compared to the bifactor model ones (Bifac-GR, 0.62–0.42 for state, 0.40–0.65 for trait).

### 3.6. Multidimensional Item Diagnostic

The mIRT analyses were conducted separately on the trait and state scales. Table 3 presents the multidimensional parameter estimates of the items, as well as the item diagnostics estimates for both the two-correlated factors (multi-GR) and the bifactor models. As found in the previous pairwise comparison, none of the state scale items were identified as misfitting at *p* < 0.05. On the contrary, item #14 (*p* = 0.004) for the trait scale was confirmed to misfit both multidimensional models.

Concerning state anxiety, all the multidimensional parameter values for the multi- and bifac-GR models overlapped with a minimal difference in the MDISC average values of 0.179 (SD = 0.255) and negligible differences in the three intercepts (MDIFF_1_ M ± SD= −0.011 ± 0.003; MDIFF_2_ M ± SD = −0.027 ± 0.013; MDIFF_3_ M ± SD = 0.044 ± 0.033). Five items (items #20, #21, #1, #12, #15) for state-somatic and two (items #5, #11) for state-cognitive factors demonstrated “moderate” to “high” multidimensional discrimination, according to Baker [49] and Hasmy [65], ranging from 1.069 (item #21) to 1.696 (item #5). The remaining five items showed “very high” discrimination parameters (MDISCR > 1.7).

Likewise, for the trait scale, a minimal difference was observed in the MDISC average values of 0.112 (SD = 0.057) and negligible differences in the three intercepts (MDIFF_1_ M ± SD = −0.004 ± 0.014; MDIFF_2_ M ± SD = −0.050 ± 0.035; MDIFF_3_ M ± SD = 0.099 ± 0.080). Six items (items #20, 21, 1, 2, 12, 15) for somatic and five (items #4, 5, 10, 11, 16) for cognitive factors demonstrated “moderate” to “high” multidimensional discrimination, ranging from 0.950 (item #20) to 1.646 (item #10). The remaining five items showed “very high” discrimination parameters (MDISCR > 1.7). To sum up, model parameters highlighted the equivalence for both multi- and bifac-GR models into discrimination among high and low levels of state and trait anxiety. 

## 4. Discussion

The STICSA is now a widely accepted measure of state and trait anxiety, although its dimensionality is a matter of interest among researchers [33,34]. Its attractiveness lies in its ability to measure both state and trait anxiety with a single measure, as well as their cognitive and somatic features. Far from the criticized Spielberg state-trait anxiety model [24,73,74], the STICSA displays good psychometric properties, including an excellent divergent validity. This feature represents an advantage for clinicians, enabling them to better discriminate between specific and common anxiety factors that share underlying processes with depression [75,76]. To date, addressing dimensionality issues represents a crucial step to score and interpret STICSA state and trait item values. Studies have relied on different conceptualization of STICSA scores, but a large part of them have supported two-dimension somatic-cognitive correlated factors for each state and trait scale [27,32,34,42]. 

Unlike past studies, strictly oriented to the classical psychometric approach, the present study aimed to disentangle the STICSA dimensionality issue by applying the multidimensional IRT approach. Multidimensional item response theory (mIRT) represents an extension over the classical IRT approach in which an additional vector of multiple personal attributes is included so that multiple underlying traits can be simultaneously measured [48,77]. Based on Styck, Rodriguez, and Yi [33]’s findings, a bifactor model was tested as a viable and reliable alternative to multidimensional models for assessing the STICSA state and trait scale dimensionality. In detail, information about fitting unidimensional, multi-GR, and bifac-GR models, testing pertinent IRT assumptions, and looking at model-data fit statistics was supplied. Indeed, that a model fits the data well overall compared to other models, but may poorly fit items, is usually observed [78]. Likewise, to interpret the bifac-GR model results, item parameters and TIFs were compared to the several models (following [50]). Parameter estimates and their multidimensional versions were computed to make meaningful comparisons across model parameter estimates (e.g., unidimensional vs. bifactor model; multidimensional vs. bifactor model) and select a championed model. 

In line with Styck, Rodriguez, and Yi [33]’s model fit, results suggested that the bifac-GR model fit our data best when compared to the multi- and uni-GR. However, when we compared the IRT conditional/marginal and multidimensional parameters estimated and the information, we observed that the STICSA bifactor model did not offer as many advantages as expected. Yet, in the present study, the restricted bifactor model offered a way to deal with violations of LD (without removing them completely), and to improve the model’s fit. It has been widely attested that a bifactor model is more robust compared to minor model misspecifications due more to unmodeled complexity than correlated factor models. It has increased parametric complexity [79] when compared to correlated factor models. Likewise, larger models tended to fit data sets better than more parsimonious models, though there is a trade-off with generalizability when models overfit a data set [39,80]. In addition, probifactor bias occurs due to the functional form of the parameters estimated in the bifactor model (e.g., [80]; i.e., the “difficulty” of the estimated parameters). Models that estimate the same number of parameters can differ in functional form, and two models with different functional forms, but equivalent free parameters, will exhibit different fitting propensities.

In the present study, the importance of estimating marginal parameters and TIF over the conditional bifactor model was demonstrated. Discrepancy among conditional and marginal slopes emerged since the specific latent-attribute did not affect the probability of response to the item (they are principally reflective of the general trait and secondarily a specific trait) [50,69]. The conditional bifactor model seemed to account for the general state/trait dimension common to all STICSA items, but it failed to address additional specific somatic/cognitive dimensions. In particular, the conditional bifactor model failed to address common variance in cognitive group item contents in the state scale and cognitive group item contents in trait scale. However, this result was already known in the STICSA psychometrics literature [33]. Small factor loadings and zero or negative group factor variances were also commonly identifiable in several studies that aimed to address a ‘general psychopathology’ reflecting commonality among all forms of psychopathology, along with several narrower psychopathology group factors (most commonly internalizing; see [81]). However, these findings can have limitations from a practical/clinical point of view. For example, the observed total/subscale scores may reflect a mixture of general and group factor variance. Observed correlations might be inflated or attenuated, since they might reflect the influence of the general factor, the group factor, or both. As a results, clinicians might be led to an incorrect assessment and treatment planning during their practices, with harmful consequences [82,83].

Therefore, it becomes mandatory to discern among broad and narrow factors to assess whether factors reflect substantive constructs or artefacts. In line with this recommendation, the usefulness of the STICSA bifactor model in computing a pairwise comparison approach was evaluated. Utilizing the estimated marginal bifactor model and parameters (also restricted), the general dimension was of the most concern, and items were allowed to load in specific factors when necessary (to account for an excess of LD). This model was found to be more precise and informative than the unidimensional and conditional bifactor models. Not surprisingly, unidimensional IRT models show a lack of measurement precision in the estimate dimensionality issue when scales are composed of correlated latent traits [84]. The computation of marginal trace line transformation also emphasized the usefulness of testing IRT model assumptions within the context of classical bifactor modeling.

Next, the suitability of a multidimensional nature of each STICSA state/trait scale was secured via IRT, and it was found that two-correlated factors represent a plausible alternative to the bifactor model, as items fit and multidimensional parameters of both multi-GR and bifac-GR were compared. We tested the possibility that a change in dimensionality of the STICSA model resulted in a different parameterization and meaning of the scale. The results of this second step showed that item #14 (“My arms and legs feel stiff”) of the trait scale displayed a poor fit for both compared models. At the parameter level, no significant differences were observed between state and trait items in both models. Compared to the cognitive ones, the somatic items were found to be less discriminative both in trait and state scales. Opposing views exist when explaining the role of either somatic or cognitive patterns of anxiety. For instance, some physiological symptom items in anxiety measures (i.e., in pain scale) have been criticized due to possible overlap with depression symptomatology and have also raised doubts about the uniformity of the anxiety construct [85]. Likewise, somatic and cognitive items were equally endorsed by psychiatric inpatients, and psychological distress was limited to somatic signs of anxiety [86]. As highlighted by different authors, the somatic-cognitive pattern of anxiety gained resonance among researchers working to ascertain the experiential components of anxiety [74,87]. Concerning the item multidimensional thresholds, in the present study, all the STICSA items encompassed a discrete range of the latent trait continuum, and also showed a clear ordering of the items based on each item’s quality and estimated level of multidimensional difficulty. 

## 5. Conclusions

Summing up, our findings supported the equivalence of the bifactor and two-factor correlated models in terms of somatic-cognitive item multidimensional parameters. When only the fit indices of the model were taken into account, the classical bifactor model outperformed our data. Despite this, the bifactor global domain (state or trait) seems to be not as accurate and precise as expected. To overcome this and other methodological issues (e.g., the LD issue within the IRT), other computations might be needed, as in the present investigation (i.e., the marginal trace line transformation). To date, single global STICSA state or trait scores should be used with caution. 

Our results showed the viability of measuring trait and state anxiety by the STICSA measure in a community sample with a wide age range, thus demonstrating its versatility. While different confirmatory factor models have been found in literature [33,34], in this study, a two-factor model for both the trait and state scales was confirmed and preferred over the inflated classical bifactor model. Specifically, IRT yielded factors that were consistent with the original domains and showed good fit on a separate set of trait and state datasets [33,34]. The moderately high intercorrelations among somatic and cognitive factors provided support for the compensatory nature of the latent traits [88]. In this way, somatic and cognitive symptoms as measured by STICSA items concurred to improve the measurement precision of anxiety. 

The results of the mIRT analysis of the STICSA trait and state scales suggested a more accurate method for evaluating the measure and, ultimately, participants’ anxiety. When the discrimination parameter was taken into consideration, items on both the trait and state scales exhibited high multidimensional discrimination values, in line with past studies’ results found in the literature on existing clinical instruments [89,90,91]. 

Further research is needed to verify this hypothesis in clinical samples. The results of this investigation suggest that somatic and cognitive symptoms are equivalent to pinpointing a stable tendency to respond to threats. The greatest advantage of applying multidimensional item response models to clinical assessment is that the symptoms may be treated as ordered indicators of risk, as well as that specific symptoms and specific factors could be scaled onto a common trait. In clinical assessment, unobservable psychopathological constructs are multidimensional in nature (e.g., mental health or well-being). This is also confirmed in the recent success of transdiagnostic treatments for anxiety and mood disorders with high comorbidity [92]. 

Other limitations of this study include sample size and characteristics. The sample involved is quite large and heterogeneous, and no data were collected regarding diagnosis. As a consequence, the nature of the response process, as well as differences in the trait manifestation, could differ among patients with anxiety as a primary diagnosis from depressed patients with anxiety in comorbidity. Future research should address the feasibility of the mIRT models in clinical samples. Likewise, from a methodological point of view, there is no consensus among researchers that the accuracy of parameter estimates in mIRT may be biased or inflated by the sample size [93,94].

## Figures and Tables

**Table 1:** Results from uni-GR, multi-GR, and bifac-GR models fit to the 21-item STICSA-STATE.

**(a)**			
	**Uni-GR**	**Multi-GR**	**Bifac-GR**
# of positive LD pairs flagged	16	8	4
# of negative LD pairs flagged	3	2	2
# of parameters	84	85	105
−2LL	−60,047.66	−58,793.31	−58,477.48
BIC	120,776.5	118,275.9	117,806.4
AIC	120,263.3	117,756.6	117,165
C2 (*df*)	6505.643 (189) ***	2499.159 (188) ***	1858.838 (168) ***
RMSEA	0.100	0.061	0.055
TLI	0.916	0.969	0.974
CFI	0.984	0.972	0.979
SRMSR	0.074	0.047	0.043
Precision (marginal reliability) [θ −3;3]	0.89	0.86–0.88	0.087–0.62–0.42
**(b)**			
	**Uni-GR**	**Multi-GR**	**Bifac-GR**
# of positive LD pairs flagged	9	6	3
# of negative LD pairs flagged	1	0	0
# of parameters	84	85	105
−2LL	−69,934.32	−69,002.42	−68,742.61
BIC	140,549.8	138,694.1	138,336.7
AIC	140,036.6	138,174.8	137,695.2
C2 (*df*)	5253.537(189) ***	2673.871(188) ***	2033.147(168) ***
RMSEA	0.090	0.063	0.058
TLI	0.919	0.960	0.966
CFI	0.927	0.964	0.973
SRMR	0.065	0.047	0.041
Precision (marginal reliability) [θ −3;3]	0.90	0.86–0.88	0.86–0.40–0.65

Note. −2LL = −2 log likelihood or deviance statistic; BIC = Bayesian information criterion; AIC = Akaike information criterion; C2 = limited-information goodness-of-fit statistic; RMSEA = root mean square error of approximation based on C2. Number of LD possible = j(j − 1)/2 = 21(21 − 1)/2 = 210. Correlation among latent traits in the multi-GR model was 0.76/0.73. *** *p* < 0.001.

**Table 2:** Unidimensional and bifactor model item slope estimates for the 21-item STICSA state and trait.

STICSA—State								STICSA—Trait							
Item	Uni-GR	Bifac-GR Conditional			Bifac-GR Marginal			Item	Uni-GR	Bifac-GR Conditional			Bifac-GR Marginal		
	a	a_g_	a_S1_	a_S2_	a*_g_	a*_S1_	a*_S2_		a	a_g_	a_S1_	a_S2_	a*_g_	a*_S1_	a*_S2_
STIC_S_1	1.196	1.017	0.979		0.881	0.840		STIC_T_1	1.020	1.205	−0.392		1.174	−0.320	
STIC_S_2	1.528	1.365	1.415		1.049	1.103		STIC_T_2	1.264	1.402	−0.078		1.401	−0.060	
STIC_S_6	1.649	1.442	1.574		1.058	1.200		STIC_T_6	1.485	1.859	0.608		1.750	0.410	
STIC_S_7	1.799	1.659	1.636		1.195	1.171		STIC_T_7	1.577	2.543	1.501		1.906	0.834	
STIC_S_8	2.516	2.389	1.993		1.550	1.156		STIC_T_8	1.991	2.565	0.746		2.349	0.412	
STIC_S_12	1.339	1.143	1.063		0.969	0.882		STIC_T_12	1.285	1.514	−0.100		1.511	−0.075	
STIC_S_14	2.012	1.910	1.928		1.263	1.282		STIC_T_14	1.801	2.118	0.154		2.109	0.096	
STIC_S_15	1.280	1.074	1.018		0.921	0.861		STIC_T_15	1.406	1.659	−0.162		1.652	−0.116	
STIC_S_18	1.817	1.597	1.177		1.313	0.858		STIC_T_18	1.723	2.025	−0.390		1.974	−0.251	
STIC_S_20	1.055	.914	0.591		0.863	0.521		STIC_T_20	0.897	1.056	−0.571		1.001	−0.485	
STIC_S_21	0.920	0.736	0.773		0.670	0.709		STIC_T_21	0.922	1.131	−0.528		1.080	−0.440	
STIC_S_3	1.980	2.207		0.148	2.207		0.090	STIC_T_3	1.691	1.443		1.332	1.443		1.266
STIC_S_4	1.715	1.816		−0.146	1.816		−0.100	STIC_T_4	1.409	1.173		1.026	1.173		0.973
STIC_S_5	1.551	1.646		0.314	1.646		0.228	STIC_T_5	1.307	1.066		1.023	1.066		1.007
STIC_S_9	1.897	2.882		−1.023	2.882		−0.546	STIC_T_9	1.669	1.431		1.253	1.431		1.161
STIC_S_10	1.534	1.933		0.601	1.933		0.408	STIC_T_10	1.426	1.182		1.242	1.182		1.274
STIC_S_11	1.298	1.191		0.143	1.191		0.117	STIC_T_11	1.030	0.965		0.335	0.965		0.296
STIC_S_13	2.256	3.932		−1.549	3.932		−0.659	STIC_T_13	1.919	1.671		1.360	1.671		1.181
STIC_S_16	1.643	1.883		0.555	1.883		0.381	STIC_T_16	1.352	1.127		0.914	1.127		0.852
STIC_S_17	2.063	2.909		1.146	2.909		0.615	STIC_T_17	1.900	1.651		1.411	1.651		1.260
STIC_S_19	2.108	2.597		0.820	2.597		0.466	STIC_T_19	1.964	1.722		1.387	1.722		1.189

Note. a = uni-GR slope; ag = conditional slope for STICSA state or trait general trait; aS1 − aS2 = conditional slopes for specific traits somatic/cognitive; a*g = marginal slope for STICSA state or trait general trait; a*S1 − a* − S = marginal slopes for specific traits somatic/cognitive.

**Table 3:** Multi-GR and bifac-GR item fit and multidimensional item parameters for the STICSA state scale.

**(a)**											
**STICSA—State**											
**Bifac-GR**						**Multi-GR**					
	**MDISC**	**MDIFF_1_**	**MDIFF_2_**	**MDIFF_3_**	**Itemfit *p* (fdr)**		**MDISC**	**MDIFF_1_**	**MDIFF_2_**	**MDIFF_3_**	**Itemfit *p* (fdr)**
STIC_S_1	1.412	0.261	1.396	2.885	0.504	STIC_S_1	1.413	0.268	1.403	2.891	0.573
STIC_S_2	1.966	0.186	1.371	2.311	0.599	STIC_S_2	1.924	0.194	1.390	2.342	0.546
STIC_S_6	2.135	0.831	1.735	2.533	0.614	STIC_S_6	2.084	0.843	1.757	2.567	0.609
STIC_S_7	2.330	0.465	1.405	2.337	0.145	STIC_S_7	2.293	0.474	1.420	2.360	0.128
STIC_S_8	3.112	0.935	1.626	2.293	0.599	STIC_S_8	3.145	0.941	1.632	2.300	0.701
STIC_S_12	1.561	0.649	1.752	2.919	0.467	STIC_S_12	1.565	0.655	1.758	2.924	0.389
STIC_S_14	2.713	0.654	1.537	2.282	0.321	STIC_S_14	2.658	0.663	1.554	2.306	0.128
STIC_S_15	1.480	0.596	1.847	2.875	0.374	STIC_S_15	1.484	0.601	1.852	2.878	0.268
STIC_S_18	1.984	0.813	1.719	2.666	0.609	STIC_S_18	2.008	0.817	1.719	2.662	0.633
STIC_S_20	1.088	1.086	2.082	3.154	0.488	STIC_S_20	1.095	1.089	2.083	3.150	0.526
STIC_S_21	1.067	0.648	1.945	3.091	0.145	STIC_S_21	1.069	0.653	1.948	3.094	0.128
STIC_S_3	2.211	−0.232	0.945	1.718	0.145	STIC_S_3	2.272	−0.222	0.944	1.708	0.144
STIC_S_4	1.822	0.462	1.505	2.446	0.488	STIC_S_4	1.822	0.469	1.510	2.455	0.473
STIC_S_5	1.676	0.052	1.323	2.253	0.145	STIC_S_5	1.696	0.059	1.324	2.248	0.114
STIC_S_9	3.058	0.380	1.206	2.006	0.145	STIC_S_9	2.161	0.421	1.336	2.258	0.131
STIC_S_10	2.024	−0.398	0.631	1.378	0.145	STIC_S_10	1.898	−0.403	0.658	1.426	0.114
STIC_S_11	1.200	0.037	1.611	2.972	0.145	STIC_S_11	1.217	0.044	1.604	2.952	0.114
STIC_S_13	4.226	0.571	1.306	1.931	0.567	STIC_S_13	2.467	0.648	1.471	2.200	0.796
STIC_S_16	1.964	−0.028	0.971	1.806	0.145	STIC_S_16	1.870	−0.024	0.998	1.854	0.114
STIC_S_17	3.127	0.027	0.981	1.761	0.479	STIC_S_17	2.526	0.031	1.049	1.881	0.268
STIC_S_19	2.723	0.124	1.070	1.797	0.437	STIC_S_19	2.456	0.131	1.117	1.872	0.276
**(b)**											
**STICSA—Trait**											
**Bifac-GR**						**Multi-GR**					
	**MDISC**	**MDIFF_1_**	**MDIFF_2_**	**MDIFF_3_**	**itemfit *p* (fdr)**		**MDISC**	**MDIFF_1_**	**MDIFF_2_**	**MDIFF_3_**	**itemfit *p* (fdr)**
STIC_T_1	1.267	−1.171	0.621	2.577	0.074	STIC_T_1	1.151	−1.239	0.664	2.757	0.055
STIC_T_2	1.405	−0.917	0.781	2.367	0.074	STIC_T_2	1.409	−0.910	0.785	2.369	0.055
STIC_T_6	1.956	0.160	1.270	2.334	0.999	STIC_T_6	1.802	0.174	1.325	2.433	0.968
STIC_T_7	2.953	−0.090	1.059	2.023	0.100	STIC_T_7	1.917	−0.097	1.227	2.365	0.042
STIC_T_8	2.672	0.528	1.450	2.217	0.044	STIC_T_8	2.444	0.550	1.499	2.288	0.016
STIC_T_12	1.517	0.204	1.477	2.735	0.999	STIC_T_12	1.490	0.211	1.498	2.772	1.000
STIC_T_14	2.123	0.501	1.537	2.514	0.004	STIC_T_14	2.154	0.504	1.534	2.508	0.003
STIC_T_15	1.667	0.264	1.619	2.744	0.102	STIC_T_15	1.631	0.271	1.641	2.786	0.117
STIC_T_18	2.062	0.381	1.499	2.506	0.796	STIC_T_18	1.891	0.397	1.558	2.623	0.770
STIC_T_20	1.200	0.138	1.453	2.803	0.044	STIC_T_20	0.950	0.168	1.713	3.332	0.037
STIC_T_21	1.249	0.331	1.695	2.846	0.696	STIC_T_21	1.024	0.380	1.943	3.286	0.630
STIC_T_3	1.964	−0.858	0.556	1.724	0.837	STIC_T_3	1.936	−0.858	0.565	1.741	0.691
STIC_T_4	1.558	−0.004	1.346	2.619	0.587	STIC_T_4	1.552	0.002	1.356	2.633	0.591
STIC_T_5	1.478	−0.322	1.067	2.353	0.308	STIC_T_5	1.464	−0.317	1.080	2.373	0.204
STIC_T_9	1.902	−0.051	1.061	2.138	0.074	STIC_T_9	1.898	−0.046	1.069	2.147	0.063
STIC_T_10	1.714	−1.056	0.235	1.345	0.117	STIC_T_10	1.646	−1.072	0.245	1.376	0.055
STIC_T_11	1.021	−0.482	1.429	3.012	0.187	STIC_T_11	0.974	−0.495	1.482	3.128	0.195
STIC_T_13	2.155	0.315	1.370	2.211	0.074	STIC_T_13	2.170	0.319	1.374	2.212	0.055
STIC_T_16	1.451	−0.526	0.760	2.023	0.074	STIC_T_16	1.448	−0.522	0.767	2.032	0.055
STIC_T_17	2.172	−0.351	0.822	1.914	0.074	STIC_T_17	2.179	−0.345	0.827	1.918	0.055
STIC_T_19	2.211	−0.154	0.914	1.842	0.385	STIC_T_19	2.215	−0.150	0.920	1.849	0.411

Note MDISC = Multidimensional Item Discrimination; MDIFF = Multidimensional Difficulty Index.

**Figure A1:**
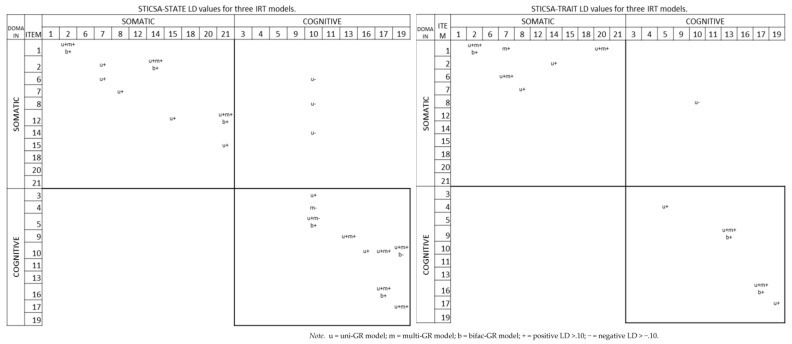
LD Indices for the Uni-, Multi-, Bifac−GR Models of the STICSA State and Trait Scales.

## Data Availability

The data presented in this study are available on request from the corresponding authors. The data are not publicly available due to privacy reasons.

## Appendix B

**Table A1:** Unidimensional and Bifactor Graded Response Model Item Parameter Estimates for the 21-Item STICSA State.

Item	Uni-GR				Bifac-GR Conditional						Bifac-GR Marginal					
	c1	c2	c3	a	c1	c2	c3	a_g_	a_S1_	a_S2_	c1	c2	c3	a*_g_	a*_S1_	a*_S2_
STIC_S_1	−0.339	−1.839	−3.855	1.196	−0.369	−1.971	−4.072	1.017	0.979		−0.363	−1.938	−4.004	0.881	0.840	
STIC_S_2	−0.314	−2.337	−4.020	1.528	−0.365	−2.695	−4.543	1.365	1.415		−0.267	−1.974	−3.328	1.049	1.103	
STIC_S_6	−1.517	−3.181	−4.715	1.649	−1.774	−3.704	−5.407	1.442	1.574		−1.230	−2.569	−3.750	1.058	1.200	
STIC_S_7	−0.924	−2.791	−4.698	1.799	−1.084	−3.274	−5.445	1.659	1.636		−0.653	−1.973	−3.282	1.195	1.171	
STIC_S_8	−2.497	−4.307	−6.115	2.516	−2.910	−5.060	−7.136	2.389	1.993		−1.218	−2.118	−2.987	1.550	1.156	
STIC_S_12	−0.942	−2.550	−4.291	1.339	−1.013	−2.735	−4.556	1.143	1.063		−0.886	−2.393	−3.986	0.969	0.882	
STIC_S_14	−1.442	−3.405	−5.131	2.012	−1.773	−4.170	−6.191	1.910	1.928		−0.928	−2.183	−3.241	1.263	1.282	
STIC_S_15	−0.824	−2.566	−4.022	1.280	−0.881	−2.732	−4.254	1.074	1.018		−0.820	−2.544	−3.961	0.921	0.861	
STIC_S_18	−1.530	−3.229	−5.039	1.817	−1.612	−3.409	−5.289	1.597	1.177		−1.009	−2.135	−3.312	1.313	0.858	
STIC_S_20	−1.168	−2.239	−3.398	1.055	−1.181	−2.265	−3.432	0.914	0.591		−1.292	−2.478	−3.755	0.863	0.521	
STIC_S_21	−0.656	−1.985	−3.180	0.920	−0.691	−2.075	−3.299	0.736	0.773		−0.939	−2.819	−4.482	0.670	0.709	
STIC_S_3	0.477	−1.958	−3.549	1.980	0.512	−2.091	−3.799	2.207		0.148	0.232	−0.947	−1.721	2.207		0.090
STIC_S_4	−0.815	−2.660	−4.337	1.715	−0.842	−2.741	−4.457	1.816		−0.146	−0.464	−1.509	−2.454	1.816		−0.100
STIC_S_5	−0.093	−2.153	−3.653	1.551	−0.088	−2.217	−3.775	1.646		0.314	−0.053	−1.347	−2.293	1.646		0.228
STIC_S_9	−0.824	−2.660	−4.519	1.897	−1.162	−3.689	−6.136	2.882		−1.023	−0.403	−1.280	−2.129	2.882		−0.546
STIC_S_10	0.701	−1.102	−2.407	1.534	0.806	−1.278	−2.789	1.933		0.601	0.417	−0.661	−1.443	1.933		0.408
STIC_S_11	−0.054	−1.999	−3.664	1.298	−0.045	−1.933	−3.566	1.191		0.143	−0.038	−1.623	−2.994	1.191		0.117
STIC_S_13	−1.492	−3.428	−5.117	2.256	−2.414	−5.521	−8.163	3.932		−1.549	−0.614	−1.404	−2.076	3.932		−0.659
STIC_S_16	0.052	−1.733	−3.247	1.643	0.055	−1.907	−3.546	1.883		0.555	0.029	−1.013	−1.883	1.883		0.381
STIC_S_17	−0.051	−2.320	−4.177	2.063	−0.085	−3.067	−5.507	2.909		1.146	−0.029	−1.054	−1.893	2.909		0.615
STIC_S_19	−0.275	−2.475	−4.163	2.108	−0.337	−2.912	−4.893	2.597		0.820	−0.130	−1.121	−1.884	2.597		0.466

Note. a = uni−GR slope; ag = conditional slope for STICSA State general trait; aS1–aS2 = conditional slopes for specific traits Somatic/Cognitive; a*g = marginal slope for STICSA State general trait; a*S1–a*−S = marginal slopes for specific traits Somatic/Cognitive; c1–c4 = intercepts.

**Table A2:** Unidimensional and Bifactor Graded Response Model Item Parameter Estimates for the 21-Item STICSA Trait.

Item	Uni-GR				Bifac-GR Conditional						Bifac-GR Marginal					
	c1	c2	c3	a	c1	c2	c3	a_g_	a_S1_	a_S2_	c1	c2	c3	a*_g_	a*_S1_	a*_S2_
STIC_T_1	1.377	−0.721	−3.059	1.020	1.483	−0.787	−3.264	1.205	−0.392		1.231	−0.653	−2.709	1.174	−0.320	
STIC_T_2	1.230	−1.044	−3.202	1.264	1.288	−1.096	−3.324	1.402	−0.078		0.919	−0.782	−2.371	1.401	−0.060	
STIC_T_6	−0.267	−2.154	−3.997	1.485	−0.314	−2.483	−4.564	1.859	0.608		−0.169	−1.336	−2.455	1.750	0.410	
STIC_T_7	0.185	−2.107	−4.112	1.577	0.266	−3.127	−5.972	2.543	1.501		0.105	−1.230	−2.348	1.906	0.834	
STIC_T_8	−1.162	−3.211	−4.927	1.991	−1.409	−3.874	−5.923	2.565	0.746		−0.549	−1.510	−2.309	2.349	0.412	
STIC_T_12	−0.281	−2.088	−3.909	1.285	−0.310	−2.241	−4.149	1.514	−0.100		−0.205	−1.480	−2.740	1.511	−0.075	
STIC_T_14	−0.961	−2.969	−4.892	1.801	−1.064	−3.264	−5.338	2.118	0.154		−0.502	−1.541	−2.520	2.109	0.096	
STIC_T_15	−0.399	−2.496	−4.260	1.406	−0.440	−2.699	−4.574	1.659	−0.162		−0.265	−1.627	−2.757	1.652	−0.116	
STIC_T_18	−0.694	−2.780	−4.702	1.723	−0.785	−3.091	−5.169	2.025	−0.390		−0.388	−1.526	−2.553	1.974	−0.251	
STIC_T_20	−0.147	−1.589	−3.112	0.897	−0.165	−1.745	−3.365	1.056	−0.571		−0.156	−1.652	−3.187	1.001	−0.485	
STIC_T_21	−0.367	−1.924	−3.278	0.922	−0.413	−2.116	−3.553	1.131	−0.528		−0.365	−1.871	−3.141	1.080	−0.440	
STIC_T_3	1.553	−1.003	−3.123	1.691	1.685	−1.092	−3.387	1.443		1.332	1.168	−0.757	−2.347	1.443		1.266
STIC_T_4	0.004	−2.006	−3.928	1.409	0.006	−2.097	−4.081	1.173		1.026	0.005	−1.788	−3.479	1.173		0.973
STIC_T_5	0.447	−1.503	−3.320	1.307	0.476	−1.577	−3.478	1.066		1.023	0.447	−1.479	−3.263	1.066		1.007
STIC_T_9	0.094	−1.883	−3.802	1.669	0.097	−2.019	−4.066	1.431		1.253	0.068	−1.411	−2.841	1.431		1.161
STIC_T_10	1.659	−0.365	−2.105	1.426	1.811	−0.403	−2.305	1.182		1.242	1.532	−0.341	−1.950	1.182		1.274
STIC_T_11	0.492	−1.463	−3.082	1.030	0.493	−1.459	−3.076	0.965		0.335	0.511	−1.512	−3.188	0.965		0.296
STIC_T_13	−0.630	−2.761	−4.466	1.919	−0.678	−2.953	−4.764	1.671		1.360	−0.406	−1.767	−2.851	1.671		1.181
STIC_T_16	0.740	−1.064	−2.849	1.352	0.763	−1.103	−2.935	1.127		0.914	0.677	−0.979	−2.604	1.127		0.852
STIC_T_17	0.705	−1.650	−3.856	1.900	0.762	−1.785	−4.157	1.651		1.411	0.462	−1.081	−2.518	1.651		1.260
STIC_T_19	0.314	−1.886	−3.806	1.964	0.341	−2.022	−4.074	1.722		1.387	0.198	−1.174	−2.366	1.722		1.189

Note. a = uni-GR slope; ag = conditional slope for STICSA Trait general trait; aS1–aS2 = conditional slopes for specific traits Somatic/Cognitive; a*g = marginal slope for STICSA Trait general trait; a*S1–a*-S = marginal slopes for specific traits Somatic/Cognitive; c1–c4 = intercepts.
